# Residential History and Groundwater Modeling

**DOI:** 10.1289/ehp.1002444

**Published:** 2010-09

**Authors:** Laurel A. Schaider, Ruthann A. Rudel, Janet M. Ackerman, Julia G. Brody

**Affiliations:** Silent Spring Institute, Newton, Massachusetts, E-mail: schaider@silentspring.org

In a case–control study, Gallagher et al. (2010) reported elevated breast cancer risk among women residing on Cape Cod, Massachusetts, who consumed water from wells classified as impacted by wastewater treatment plant effluent from the Barnstable Water Pollution Control Facility (BWPCF). Exposure classification was based on a U.S. Geological Survey model of groundwater flow, historical public water supply data, and the women’s residential histories. Recent detection of hormones and other wastewater-associated endocrine- disrupting compounds (EDCs) in drinking water supplies nationwide has raised concerns about health effects, so we applaud the authors for an inventive examination of this issue, which is difficult to study because historical exposure measurements are lacking.

However, drinking water data for Cape Cod suggest additional exposures that are relevant to this analysis. An alternative, or possibly additional, explanation for the elevated breast cancer risk may be drinking water exposures to volatile organic compounds (VOCs), some of which are mammary gland carcinogens (Rudel et al. 2007). Although the Barnstable Water Company (BWC)—which provided water to exposed participants—included three wells impacted by the BWPCF, it also included three wells contaminated by VOCs from a nearby airport. Treatment was installed in 1991 (Keijser H, personal communication), so for 25 of the 27 years of the study’s exposure period (1966–1993), there was no treatment to remove VOCs. Contributions of these VOC-contaminated wells to the BWC system were similar to, and in some cases exceeded, the contributions from BWPCF-impacted wells. In 1975, the wells impacted by the airport (Maher wells 1, 2, and 3) supplied 43% of BWC water, compared with 41% from the wastewater-affected wells (Hyannisport, Simmons Pond, and Straightway) (LeBlanc et al. 1986); in 1986, the proportions were 33% and 37%, respectively (Bratton 1991). In addition, at least one BWPCF-impacted well also contained VOCs, as Gallagher et al. (2010) mentioned.

An unspecified number of BWPCF-exposed participants relied on private wells, which were probably not affected by the airport. However, from 1970 to 1990, only around 10% of homes near the BWPCF relied on private wells (Silent Spring Institute 1997), so most of the BWPCF exposures occurred through the public supply that was impacted by airport-related VOCs.

A further complication is that Gallagher et al. (2010) stated that they “determined that the BWPCF was the only source of wastewater effluent with the potential to impact the drinking water of this study population”; however, we doubt that. Drinking water supplies of participants in both the exposed and nonexposed groups likely contained wastewater contaminants originating from septic systems. Among the exposed group, discharges from septic systems contributed substantially to the volume of water pumped from two of the BWPCF-impacted wells (Barlow 1994). Wells serving nonexposed participants also were impacted by septic systems. From 1972 to 1985, over half of the public wells in the entire study area contained elevated nitrate concentrations (Silent Spring Institute 2002). In addition, the largest source of groundwater nitrate on Cape Cod is wastewater from septic systems (Brody et al. 2006 and references cited therein).

Despite uncertainty about the composition of drinking water exposures, Gallagher et al. (2010) did provide evidence that drinking water in this area may have been associated with breast cancer. Their study highlights the value of routine tracking of drinking water quality and supports further epidemiologic breast cancer research on EDCs and VOCs in drinking water.
